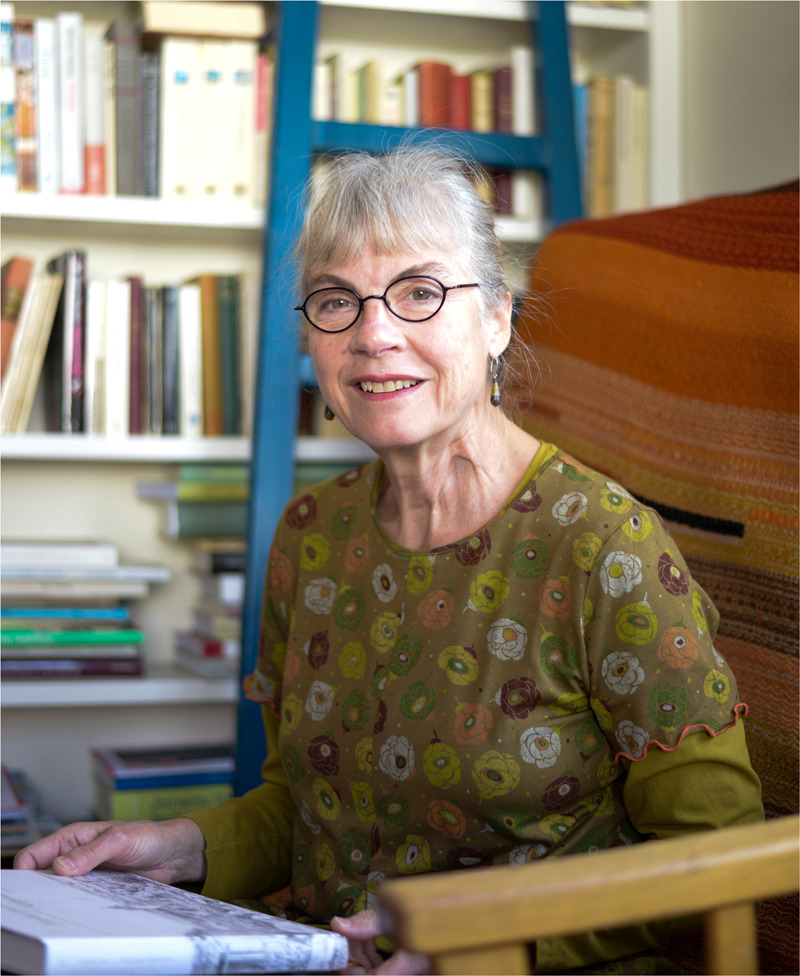# In Memoriam Kerstin Stenius 1951–2025

**DOI:** 10.1177/14550725251397286

**Published:** 2025-11-21

**Authors:** Matilda Hellman, Christoffer Tigerstedt, Johan Edman, Thomas F Babor, Mats Ramstedt, Kristiina Kuussaari, Airi Partanen, Tuukka Tammi, Pia Rosenqvist, Jessica Storbjörk, Matilda Wrede-Jäntti, Jani Selin, Margaretha Järvinen, Kim Bloomfield

**Affiliations:** 1 Department of Sociology, 225313 Uppsala University Department of Sociology, Uppsala, Sweden; 2 3837 Finnish Institute for Health and Welfare, Helsinki, Finland; 3 Department of Criminology, 7675 Stockholm University, Stockholm, Sweden; 4 Public Health Sciences, 12227 University of Connecticut School of Medicine, Farmington, CT, USA; 5 7641 Swedish Council for Information on Alcohol and other Drugs, Stockholm, Sweden; 6 Promotional and preventive work, 3837 Finnish Institute for Health and Welfare, Helsinki, Finland; 7 691868 Nordic Welfare Centre, Stockholm, Sweden; 8 Department of Public Health Sciences & Centre for Social Research on Alcohol and Drugs (SoRAD), 7675 Stockholms Universitet, Stockholm, Sweden; 9 Social Sciences/ Social Work, 743742 University of Helsinki, Helsinki, Finland; 10 Drugs and addictions, 3837 Terveyden ja hyvinvoinnin laitos, Helsinki, Sweden; 11 4321 University of Copenhagen, Copenhagen, Denmark; 12 1006 Centre for Alcohol and Drug Research, Aarhus Universitet, Copenhagen, Denmark

With immense sorrow, we received the news in August that the leading figure of the journal, Kerstin Stenius, had passed away. Kerstin had been involved with the journal since its founding in 1984, first as editor, and, in 1999, she became editor-in-chief, a position she held until the beginning of 2017. For many of us, Kerstin's academic profile was above all shaped by her great commitment to the journal. Kerstin Stenius had an outstanding career as a researcher and was a key figure in several international networks. She chaired the International Society of Addiction Journal Editors (ISAJE) and the Kettil Bruun Society for Social and Epidemiological Research on Alcohol (KBS), and she was a founder and board member of the International Confederation of ATOD Research Associations (ICARA).

When the news of Kerstin's passing was announced on the KBS and ISAJE lists, the messages expressed deep sadness over the loss of a respected and beloved colleague. She was remembered for her warmth, intelligence, generosity and major contributions to global collaboration and mentorship, especially in supporting scholars from underrepresented regions. Kerstin's roles as KBS President and founding member of ISAJE were pivotal, and many around the world recalled her kindness, humor and lasting impact on the field.

In this text, colleagues and friends share personal memories and professional insights that together form a rich portrait of Kerstin's life and legacy, touching upon her editorial vision and leadership, her mentorship and support, her ethical compass and advocacy, her work as a researcher, as well as her personal warmth and generosity. In line with her vision of a multilingual journal, some contributions are written in English and others in Swedish.

## Christoffer Tigerstedt:

In the beginning was a thin copy called *Alkoholpolitik* (i.e., a shortened Swedish translation of the Finnish journal *Alkoholipolitiikka*), published by the Finnish state alcohol monopoly. Kerstin, who was excited by Finnish alcohol research(ers), got a vision: that tiny reproduction could be developed into a creative journal. And so, she started turning a modest national publication into a Nordic journal with original articles, columns, debates and interviews, engaging researchers both within and outside the alcohol and drug field. Four dense issues per year were released throughout the 1980s.

But, Kerstin thought, this is not enough, we need more. More issues and more “Nordicness”. And so, she set out raising money from Sweden and Norway and extended the annual number of issues to six. Good timing, because shortly thereafter, in the early 1990s, Finland's, Norway's and Sweden's negotiations for Europen Union membership overshadowed political decisions based on Nordic frameworks.

Kerstin, who certainly realized that the Nordics were partly being put aside, concluded: We need readers from other parts of the world; we need a non-Nordic language!

And so, she added a seventh issue, an English supplement. This first step in anglicizing the journal brought Kerstin closer to the international community of journals. She started looking critically at the publishing policies and procedures of journals in the field and comprehended: We need more ethical thinking, and we need better writing!

Kerstin became an active member of the International Society of Addiction Journal Editors (ISAJE) resulting, among others, in the book *Publishing Addiction Science: A guide for the perplexed* (North-Holland Pub Co., Amsterdam).

Gradually international collaboration and the English language got a grip on Kerstin. The English supplement was swelling threateningly. Kerstin never opposed publishing in Danish, Norwegian or Swedish, but she had a vision: We need to be more competitive; we need an international publisher!

And after prolonged negotiations *Nordic Studies on Alcohol and Drugs* (NAD) got an impact factor and a new publisher, first De Gruyter and since the beginning of 2017 SAGE. As a result, 90% of the content of NAD is now published in English.

The NAD journal was Kerstin's life's work. There are people who aim higher, who cry for the moon and even reach for the stars. Kerstin was one of them. We were strengthened by her determination and purposefulness. We would have needed her several years ahead.

## Johan Edman:

För en historiker med ett intresse av den nordiska missbruksvården var Kerstin med sitt stora kunnande om denna, och med sitt intresse för det historiska perspektivet, en ständig inspiration. Kerstin var opponent på min doktorsavhandling och ett stort stöd efter disputationen. Det var hon som hjälpte mig att få en postdoktjänst i Helsingfors efter disputationen, hon som föreslog och drev att vi tillsammans skulle vara redaktörer för en antologi om den nordiska missbruksvårdens historia och hon som bjöd in mig till seminarier och konferenser.

Det var lätt att bli vän med Kerstin som också gärna bjöd hem en till hennes och Henriks hem. Året efter min postdoc i Finland var jag exempelvis tillbaks med min fru och nyfödda dotter, självklart inbjuden till hennes och Henriks vackra landställe. Kerstin var en genuint snäll och generös människa, en fantastisk kombination av klokskap, professionalitet och värme som med en självklarhet hjälpte andra människor, inte minst mig i början av min akademiska karriär. Att skriva akademiska texter ihop med Kerstin, vilket jag hade nöjet att göra flera gånger, var därför till lika delar intellektuellt stimulerande och bara oerhört trevligt.

## Tom Babor:

Kerstin was revered by her friends and family as an extraordinary person, one whose moral compass was always pointing in the right direction. In the numerous editorials she wrote for the NAD journal, she spoke out about the threat of conflict of interest and the need for ethicality, inclusiveness and international collaboration in addiction science. When others took a wait-and-see attitude toward injustice and conflicts of interest, Kerstin was at the barricades, using her pen as a sword to stand up for addiction science.

She was as much of a human dynamo in her family and community as she was in her professional sphere. Such a full and active life stands as an example to the addiction science field she did so much to build into a global movement. When the final bell rang as she was laid to rest, it was not necessary to ask for whom the bell tolls. It was tolling for us.

## Mats Ramstedt:

I first came into contact with Kerstin Stenius in the spring of 1999, when the NAD journal was about to publish a supplement featuring articles from the ongoing ECAS project, within which I would later complete my PhD. I don’t remember all the details, but I was a bit delayed and stressed about my article, so we had a phone conversation. That conversation really calmed me down — Editor-in-Chief Kerstin was very supportive and understanding, and together we made a plan for how to complete the article. It became my first published paper, and I always associate it with Kerstin and the conversation we had.

Over the years, we met through many Nordic collaborative projects and at international KBS conferences. Around 2007, we also became colleagues at SoRAD, Stockholm University, after Kerstin had been appointed guest professor there. Her broad expertise and deep interest in alcohol and drug issues, combined with a warm and inclusive personality, were a great asset to our workplace. I remember her with warmth and as a truly wonderful colleague.

## Kristiina Kuussaari and Airi Partanen:

Kerstin's expertise in substance use services research could be described in the following key words: system orientation, integration, new divisions of labour, governance, social policy, especially Nordic comparative research and wide international networks. Besides being a widely respected researcher, she also referred to her first profession as a social worker which had created the basis for her perception of humanity.

For us, Kerstin was not only a colleague, but also a dear friend. Her humanity, her hospitality, her persistence and her true interest into people are qualities and values for which she will be remembered. She was also an excellent cook. Indeed, the most cherished of memories are the moments we were able to share around delicious food and interesting discussions in her beautiful home.

## Tuukka Tammi:

I feel truly lucky to have worked closely with Kerstin for several years. I first met her as a doctoral student – initially through the NAD journal, and later more closely in our joint research project *Substances in the Mind* (*Päihteet mielessä* in Finnish), which explored how substance use and mental health services could be better integrated, or could they. After we received funding from the Research Council of Finland, Kerstin brought me with her to the newly established THL.

That project captured much of what Kerstin had been developing for a long time together with many colleagues: a systems approach to understanding substance use treatment. It offered a refreshing and much-needed alternative to the dominant focus on individual and medicalized models. Kerstin helped us see that effective treatment is not only about methods or individual clients, but also about the broader systems that surround them — including the differing professional orientations, in this case particularly between social work and psychiatry, and the growing influence of market logic. These forces shape what treatment looks like, who receives it, and even how professionalism itself is defined within the field.

Kerstin had a gift for seeing the bigger picture – for reminding us that in understanding addiction, context matters as much as individuals.

Thank you, Kerstin – a deeply knowledgeable, curious and warm-hearted person – for everything you shared as a colleague, mentor, and friend. You are greatly missed.

## Pia Rosenqvist:

As I knew Kerstin for most of my adult years my memories of her are filled with a lot of fantastic “clips” – from working together, from celebrating different occasions with our families and friends, from travelling around in Finland and in the Nordic countries among others. There is also a lot that could be said to describe Kerstin as a person and friend, but now I'd shortly like to focus on three E’s describing at least something of her as a person.

*Energy:* Kerstin was very energetic; she had a unique capability of doing things, not leaving things in the middle and most of all a unique capability of transforming ideas into concrete action. We all also know that she used this capability in a variety of fields of life – both work and social life.

*Ethics:* To make ethically right choices, to distinguish right from wrong, good from bad, seems to have been inherent in Kerstin; she based her values on ethical principles rather than short term or easy solutions. In a way, this meant that I could always rely on her and actually be guided by her in some difficult situations.

*Empathy:* Kerstin was a very warm person. We did like each other very much, which was a strength in general but also in many job-related situations. However, she had this special kind of reacting emphatically in a variety of situations, she seemed to understand people who were not necessarily very close to her, in a way she could express their thoughts and feelings and she did not automatically dismiss reactions by others which she herself would not have had.

## Jessica Storbjörk:

It is with deep sorrow that I say farewell to Kerstin. It has been 2 months since she passed away, yet I still have not grasped the reality of it. For me, Kerstin is still here.

Perhaps this is because Kerstin has been part of almost my entire academic life. My journey began in June 2000, and Kerstin joined the Centre for Social Research on Alcohol and Drugs (SoRAD) in that same year – long-awaited for her experience. The work with Women and Men in Swedish Alcohol and Drug Treatment had started, and Kerstin was invaluable in launching the fieldwork within social services.

Her research focus then, as later, centered on groups who, in addition to alcohol or drug problems, faced other adversities, such as difficult social circumstances. She always stood on the side of the vulnerable – whether addressing alcohol problems among Finnish-born people in Sweden or discussing profit interests.

Already during Women and Men in Swedish Alcohol and Drug Treatment, Kerstin's interest in the privatization of health and social care was catching, and we followed the unfolding privatization of a large substance use unit – something still quite new in the field of substance use treatment.

Kerstin was an exceptionally likeable person – warm, positive, and supportive, yet at the same time intellectually sharp and highly driven. Many have experienced her generosity towards younger colleagues, while she navigated the most distinguished academic settings with ease. I greatly appreciated her pragmatic and solution-oriented approach. She valued fairness and equal opportunity, insisting that efforts should be recognized – such as through just authorship practices.

When Kerstin passed away, I recalled a vivid memory of us sitting by a hotel window overlooking a canal and talking about academia and life. “Kerstin, a dear colleague and friend”. I had written next to her photograph from this conference trip to Amsterdam in 2002. My photographs reveal that our first stop had been a coffee shop. The fact that 26 years separated us in age is hard to grasp; she could have been my mother, yet felt like a dear friend whose wisdom gently guided the way forward.

Kerstin placed great importance on collaboration and international comparison. In 2006, she invited me to STAKES (which later morphed into the Finnish Institute for Health and Welfare) and the then Nordic Council for Alcohol and Drug Research. I got to stay in her home, and I cherish the many occasions we shared together in different locations. She also introduced me to the world of academic publishing as co-editor of a special issue of the NAD journal.

Our paths continued to cross through shared interests and research projects, and we had the privilege of working together on studies of compulsory care, treatment systems (which gave rise to fond memories from California), new public management, and the privatization of residential care in the Nordic countries.

Kerstin devoted much of her energy to research, and I am pleased that she came to focus on her grandchildren, painting and choir singing. I am equally pleased that she joined our Nordic comparative research conference in 2024. It felt unthinkable to organize such an event without the queen of Nordic alcohol and drug research.

The world is paler without Kerstin. Yet I remain deeply grateful to have had her as an invaluable role model and enduring source of inspiration, both as a researcher and as a person. My memories of her make it clear how her warmth and dedication always reflected a profound commitment to a better society, both through her research and through the generosity and encouragement she extended to others.

## Matilda Wrede-Jäntti:

Kerstin was a knowledgeable, analytical, wise and at the same time inclusive and truly caring person. She is the best boss I ever had; trusting and supportive yet clear and sincere. I don't think many employees have spent as many enjoyable moments in their boss's home and kitchen as those she was responsible for. She was a lovely person; positive and so easy to like.

## Jani Selin:

Kerstin was unique – kind, warm-hearted, and unwaveringly supportive, especially toward younger colleagues. Her encouragement and generosity left a lasting mark on the careers of many researchers. Her legacy endures in the thought and research of many.

## Margaretha Järvinen:

I have known Kerstin for more than 40 years. One of the first things we did together was to interview doormen at restaurants and bars in Helsinki about female pub guests who had historically been banned from entering pubs and bars without male company. We had a lot of fun visiting these pubs and talking to staff members who could tell us about historical times, and about women drinking too much or otherwise behaving in inappropriate ways. We wrote an article about this, published in Alkoholpolitik 1985:1. In the many decades that I have been living in Denmark, Kerstin has visited us now and then, and we have met at international conferences and seminars and spent time together going for long walks, visiting historical sites, etc. The last time I saw Kerstin was in July 2025 when we visited Kerstin and Henrik in their beautiful home in Helsinki. Kerstin had just gone through a tough treatment process but was in a good mood and as lively and attentive as ever. I miss Kerstin for her wonderful personality, her intelligence and engagement, her warm smile, and contagious laughter.

## Kim Bloomfield:

Like many of my colleagues I am incredulous that Kerstin is gone – and much too soon. We are all still missing her greatly and grieving. It is difficult to try to write something that does justice to the beautiful person that she was.

I believe that Kerstin and I must have started our international careers about the same time. I met Kerstin when I joined Klaus Mäkelä's international study of Alcoholics Anonymous in 1988. Through this project and through beginning my membership in the Kettil Bruun Society, she and I would meet rather regularly over the following six years. We would continue to see each other at the annual KBS symposia and thematic meetings. After I moved to Denmark in 2001, there were more opportunities to meet at Nordic research meetings and during the eight-year period that I was on the editorial board of the NAD journal.

Although we had a type of regular contact, Kerstin and I were not close colleagues or friends. There were probably barriers to that. Even though Kerstin spoke incredibly fluent English, and thus we could communicate easily, I was not a true member of the Scandinavian or Nordic cultural world. We also had separate research interests and traditions. Kerstin focused on studying treatment systems and policies and approached her research from a qualitative perspective. I was involved with alcohol epidemiology, public health research and took a quantitative research approach. Yet our careers developed in parallel and at times our paths were on a “collision course” of sorts. We were both candidates for the KBS presidency at the same time, with Kerstin winning the vote during my first try. We also were both applicants for a position for a professorship at SoRAD to which Kerstin was appointed. Although I could have considered her my nemesis or my competitor, I think I rather felt admiration for her as she was always kind, charming and caring. She carried herself with grace, wit and much candid honesty.

I could not focus on her presidential speech at 2009 KBS conference dinner in Copenhagen since I was co-organiser, making sure that dinners were being served on time. But she had everyone in stitches with her comedic humour which she could convey very easily in English. At that dinner and on other occasions, one could see that Kerstin really loved to laugh and have a good joke. I can just see now, how she would move her head down slightly, then throw it back with her hair following and sort of squint at the others when she enjoyed a good laugh. It would disarm everyone at the table or in the room.

The last time I saw Kerstin was at the *Nordic Comparative Research in Addictive Substances and Behaviours* meeting at Stockholm University in April 2024. During one of the breaks when we first greeted each other, I lamented to her of the recent death of a dear colleague of ours, Austrian sociologist Irmgard Eisenbach-Stangl. We three (and other dear international colleagues) went back to the International AA study of the late 1980s. I lamented Irmgard's passing and said that I could not believe it and that it was hardly acceptable to me that Irmgard was already gone! Kerstin basically replied: “Well, we all die, Kim. That's life”. Again, the candid honesty of Kerstin which I – and we – must now accept.

## Matilda Hellman:

Kerstin anställde mig som vetenskaplig redaktör år 2003. Året innan hade jag tagit min politices magisterexamen och blivit mamma. Det var stort att bli intervjuad för ett jobb vid *Forsknings- och utvecklingscentralen för social- och hälsovården (Stakes)*. Under arbetsintervjun frågade hon om jag själv var intresserad av att forska. Det svarade jag att jag var. När hon efter intervjun följde mig ut från kontoret på Fågelviksgränden i Helsingfors, tittade hon upp mot de tunga grå molnen och konstaterade glatt: “Det har ännu inte kommit något regn”.

Att just dessa två saker dröjt sig kvar i mitt minne från intervjun – en omfattande, prövande fråga och en lättsam, omtänksamt social kommentar – är på något plan beskrivande. Hon var en slags kraftpunkt där skärpa och värme möttes.

Kerstin var den första i en rad kvinnor som kommit att forma och bekräfta min akademiska habitus. Jag kände mig främmande inför formaterad stel akademiskhet, men Kerstin visade att man kunde vara sig själv i alla lägen: personlig, yvig, och suverän och välja följsamhet enligt strikta vetenskapliga kriterier utan att underminera sina personliga styrkor. Hon var verkligen en frisk intellektuell fläkt i Stakes annars inrutade och något tröga arbetsmiljö. Det här också på ett rent fysiskt plan: med sin rikssvenska accent när hon talade finska och rörde sig kvickt i korridorer och i möteslokaler, hur hon presenterade sig själv. Ständigt ödmjuk och snäll men på samma gång beslutsam och handlingskraftig.

Kerstin var också en sällsynt blandning av stort allvar och stor humor. När det gällde det viktiga, som att stå upp mot nyliberalistiska vansinnigheter, var hon kompromisslöst arg och seriös. Men allvaret kunde brytas i fullkomlig kontrast till en slags observerande absurd humor. När jag talade med henne i telefon i somras uttryckte hon både mod och realism beträffande den sjukdom som präglade hennes sista månader. Men sedan ville hon byta samtalsämne och härmade skrattande de olika röstlägen som hon fått öva på sin röstlektion.

Mitt finaste minne av Kerstin när hon hösten 2023 kom till Uppsala för att närvara vid min professorsinstallation. Då vi tågade ut ur universitetets aula hade vi framför oss ett hav av människor, men jag såg genast hennes lilla gestalt i lång kappa med det kännspaka glasögonbeklädda ansiktet. På middagen höll hon sedan ett briljant och hysteriskt roligt tal och vann med lätthet alla närvarandes fullfjädrade beundran.

Det har varit ett enormt privilegium att ha fått ha Kerstin som en ledstjärna för min utveckling. Hon förblir ett starkt inslag, men kommer alltid att fattas mig.